# Stable Isotope Analysis for the Discrimination of the Geographical Origin of Greek Bottarga ‘Avgotaracho Messolongiou’: A Preliminary Research

**DOI:** 10.3390/foods11192960

**Published:** 2022-09-22

**Authors:** Anna-Akrivi Thomatou, Eleni Psarra, Eleni C. Mazarakioti, Katerina Katerinopoulou, Georgios Tsirogiannis, Anastasios Zotos, Achilleas Kontogeorgos, Angelos Patakas, Athanasios Ladavos

**Affiliations:** 1 Department of Business Administration of Food and Agricultural Enterprises, University of Patras, 30100 Agrinio, Greece; 2 Department of Biosystems Science and Agricultural Engineering, University of Patras, 30200 Messolongi, Greece; 3 Department of Agriculture, International Hellenic University, 57001 Thessaloniki, Greece

**Keywords:** food authenticity, geographical origin, bottarga, isotope ratio mass spectrometry (IRMS), PDO

## Abstract

Consumers are increasingly interested in the geographical origin of the foodstuff they consume as an important characteristic of food authenticity and quality. To assure the authenticity of the geographical origin, various methods have been proposed. Stable isotope analysis is a method that has been extensively used for products such as wine, oil, meat, while only a few studies have been conducted for the discrimination of seafood origin and especially for mullet roes or bottarga products. Analysis of the stable isotopes of C, N and S of Bottarga samples from four different origins were carried out. The values of *δ*^15^N (5.45‰) and *δ*^34^S (4.66‰) for the Greek Bottarga Product named ‘Avgotaracho Messolongiou’, from Messolongi lagoon were lower than other areas while *δ*^13^C values were higher (−14.84‰). The first results show that the stable isotopes ratios of carbon, nitrogen and sulphur could be used to discriminate the Greek Protected Designations of Origin Bottarga product ‘Avgotaracho Messolongiou’ from other similar products.

## 1. Introduction

In the past decade, an increased interest in high-quality foods and products was seen. Consumer demand governs the business strategies in the marketplace. Currently, there is an increasing interest in the geographical origin—authentication of the commodities. There are various reasons for this; the support of local business; the decreased consumer confidence in the quality and safety of foods; the laws of European Union (EU); and the imports of unidentified products [1]. The global commerce and the growing demand for high-quality products have led to fraudulent activities in food industry [2]. In addition, products from highly regarded geographical origins can be sold at remarkably higher prices than others coming from different or unknown provenances. For all the above reasons, it is important for food authorities to develop and adopt mechanisms to check the origin of food products.

The need for the determination of food authenticity led the EU to institute a Traceability Regulation (178/2002/EC). It came into force in January 2005 and defines the ‘food and feed traceability’. Moreover, European laws EC N. 510/2006 and 1151/2012 prevent any attempt of mislabelling concerning products with Protected Geographical Indications (PGI), Protected Designations of Origin (PDO) and Traditional Specialties Guarantee (TSG) [3]. In addition, European regulations concerning the labelling of food products (Reg Eu 1169/11) and, in particular, concerning the mandatory information to be reported on the label of fish and seafood products (Reg 1379/2013) have as scope the establishment of a common organisation of the markets in fishery and aquaculture products. However, these regulations do not point out the appropriate mechanisms for each specific food product for food authorities to be able to check for food fraud and mislabelling.

Thus, many researchers have proposed different approaches that should be evaluated for each food product. Amongst the proposed methods, the isotopic ones have been shown to be an efficient tool against the adulteration of agri-food products. Stable isotope analysis gives information about geographical origin of various food products, such as juice, wine, milk, honey, oil and meat [4,5,6,7,8,9]. Food products have a unique constitution of isotopes, which means that carbon and nitrogen isotopes are defined by the composition of animals’ nutrition, oxygen and hydrogen isotopes from climatic conditions (air, precipitation), latitude, elevation and sulphur isotopes from soil structure [10]. Aquatic foods are a substantial part of the food commerce. They are composed of omega-3 fatty acids, vitamins, minerals, trace elements, high quality protein and essential amino acids; thereby, they improve nutrition, health and well-being [11]. According to our knowledge, authenticity of aquatic food products is concerned about production method, geographical origin and biological species [3]. Few studies have been conducted on isotopic analysis for the determination of seafood, and most of them deal with the discrepancy between farmed and non-farmed products [12,13,14,15,16,17,18,19,20,21,22,23,24,25,26]. Turchini et al. (2009) [27] studied the discrimination between farms in different geographical areas by using *δ*^13^C, *δ*^15^N and *δ*^18^O isotopic ratios and concluded that the combination of these ratios could help in the differentiation of the seafood origin. Kim et al. (2015) [8] also used stable isotope analysis for the discrimination of shrimp and hairtail fish and found that *δ*^13^C and *δ*^15^N ratios can be used to differentiate the geographic origin of the same samples. Gopi, et.al. showed that isotopic profile can be used to trace the geographic origins of farmed and wildcaught Asian seabass [25]. Camin et. al. studied isotope ratio in fat and defatted fillet of 130 rainbow trout, reared with feed incorporating a high or low fish content in 20 Italian farms, focusing on two northern Italian regions. The C, N and S isotope ratios of feed and fillet were highly positively correlated with both within each matrix (feed or fillet) and between the two matrices [28]. Kang et al. (2021) determined the stable isotopic ratios of sea cucumbers from different regions of China and used different statistical models to discriminate geographical origin [29].

The mullet (*Mugil cephalus*) is a fish species that can be found in littoral waters. Roes come from the mullet’s eggs which are collected from the fish and after salting and drying treatment, a seafood product. This product is named alternatively according to the area where it is produced as Greek *Avgotaracho*, Japanese *Karasumi, Taiwanese ‘wuyuzi’* or Italian *Bottargo* [30]. Mullet Roes samples have been mainly studied concerning the characterisation of lipid fractions [31,32,33] but only a few studies have been carried out for the physicochemical characterisation of roes [34,35] and the discrimination of mullet roes of various geographical areas [34]. The mullet Roe coming for Messolongi is registered since 1996 as Protected Designations of Origin product (file name PDO-GR-0446) under the name ‘Avgotaracho Messolongiou’. The product is very important for the local economy, providing that it comes from wild-caught mullets in small quantities (about 1000 kgr per year), which increases its price to more than 400€ per kgr, contributing to fishermen’s income of the region. On the other hand, these increased prices and the low production volume motivates adulteration and fraud.

The aim of the present work is to study the application of stable isotope analysis of N, C and S using EA-IRMS in order to discriminate the geographical origin of bottarga samples from four different origins. To date, no studies have been carried out on the stable isotope composition of the Greek Bottarga, and therefore, these are the very first data on the mullet roes produced in Greece.

## 2. Materials and Methods

### 2.1. Sampling

**Figure 1:**
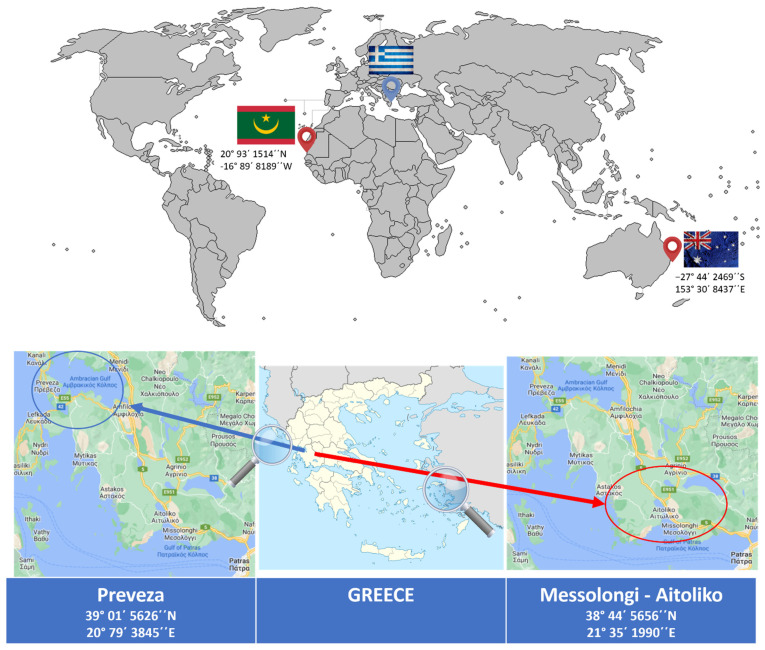
The areas producing the examined Bottarga products.

**Figure 2:**
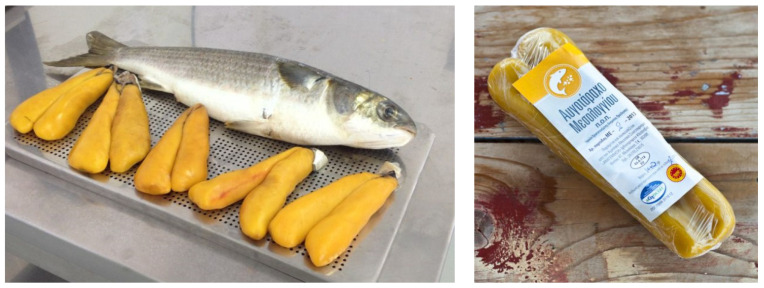
Greek Bottarga (Fish—Roe) from Messolongi—Aitoliko Lake Registered ‘Avgotaracho Messolongiou’ PDO.

The bottarga samples come from four different regions: two of them from Greece (Figure 1) namely Messolongi and Preveza. Both these areas produce Bottarga products; however, only Bottarga originated from Messolongi (Figure 2) is registered since 1996 as Protected Designations of Origin product (file name PDO-GR-0446) under the name ‘Avgotaracho Messolongiou’, as already mentioned. The rest of samples come from two other regions of the world (Australia and Mauritania). The samples were labelled by codes ‘A1’ to ‘A4’, see Table 1. In total, ten samples from Messolongi were examined and two samples from each other area were used for the stable isotope analysis. The samples of the examined areas come from the same bottarga producer and were produced during 2021. Different samples come from productions with different LOT numbers.

### 2.2. Sample Preparation

The bottarga samples (about 100 gr of every sample) were homogenised in a mill (pulverisette 11, Fritsch GmbH Milling and Sizing) and separated in two parts. For the determination of *δ*^13^C, the homogenised sample was dried in an oven at 50 °C for 24 h. For the determination of nitrogen and sulphur isotopic ratios (*δ*^15^N and *δ*^34^S), the homogenised samples were extracted with chloroform to remove lipids that can affect the determinations. Briefly, about 50 gr of homogenised sample was dispersed in 200 mL of chloroform and agitated with a magnetic stirrer for 2 h. After centrifugation, the solid part of the mixture was collected and dried in an oven at 50 °C for 2 h and then was further homogenised using a mortar. Samples were stored in freezer prior to the IRMS analysis. A previous research [36] on mullet roes in Taiwan showed there is no need to analyse different parts of the ovary (roe) as their results suggested that the isotopic compositions were homogeneous in each part of the ovary (roe) and the subsample collected from any part of the ovary (roe) can represent the isotopic values of the whole roe. The same authors [36] also concluded that there is no significant difference when they examined salted and fresh roe. Based on these suggestions no further treatment was made about salinity and the specific part of the roe.

### 2.3. EA-IRMS Analysis

The isotopic analyses of carbon, nitrogen and sulphur were performed by an Elementar Isoprime 100 Isotope Ratio Mass Spectrometry (IRMS) instrument (IsoPrime Ltd., Cheadle Hulme, UK) coupled to an elemental analyser (Elementar Vario Isotope EL Cube, Elementar Analysensysteme GmbH, Hanau, Germany). Samples ≈ 1–2 mg were weighed into tin capsules for measurement and were loaded onto the auto-sampler of the IRMS analyser. The results of the isotope ratio analyses were expressed as delta values *δ* (‰) and calculated according to the following equation:

*δ* Χ (‰) = [(R_sample_/R_standard_)^−1^] × 1000 (1)

where X is the isotope being studied (e.g., ^13^C, ^15^N, ^34^S), R_sample_ is the isotopic ratio of the measured element in its physical form (e.g., ^13^C/^12^C, ^15^N/^14^N, ^34^S/^32^S) in the sample, and R_standard_ is the isotopic ratio of the reference material.

Calibration of the IRMS instrument is required prior to sample analysis. The instrument is calibrated by using reference materials of known composition (standard substances). Depending on the isotope is needed to be determined (C, N, S), the appropriate standard substance is selected. The reference material is selected based on its physicochemical properties, i.e., it should have similar isotope ratio as the ones which are under consideration.

The analysis proceeds in a batch process by which a reference is analysed followed by a number of samples and then another reference. The reference material used for δ^13^C analysis of our samples was IAEA-600 (Caffeine, δ^13^C_V-PDB_ = −27.77‰). B259 (Sorgum flour IRMS Standard, by elemental microanalysis δ^13^C_V-PDB_ = −13.78‰) was measured as quality control check sample during analysis of our samples. The reference material used for δ^15^N and δ^34^S analysis of our samples was B2159 (Sorgum flour IRMS Standard, by elemental microanalysis δ^15^N_Air_ = 1.58‰ and δ^34^S_V-CDT_ = 10.11‰). B2155 (protein IRMS Standard, by elemental microanalysis δ^15^N_Air_ = 5.83‰ and δ^34^S_V-CDT_ = 6.18‰) was measured as quality control check sample during analysis of our samples.

## 3. Results and Discussion

### 3.1. Stable Isotope Analysis

Ten samples from Messolongi Region and two samples from the other (Preveza—Greece, Australia and Mauritania) were analysed. For each different bottarga sample, four (4) repetitions were performed. The mean values of *δ*^15^N_AIR_ (‰), *δ*^13^C_V-PDB_ (‰) and *δ*^34^S_V-CDT_ (‰) for the whole area are presented in Table 1.

Table 2 presents the results of the one way ANOVA, where it is shown that the mean values of *δ*^15^N_AIR_ (‰), *δ*^13^C_V-PDB_ (‰) and *δ*^34^S_V-CDT_ (‰) are statistically different for the examined areas.

As it can be observed, the mean values of *δ*^15^N and *δ*^34^S from Messolongi samples were significantly lower (5.45‰ and 4.67‰, respectively) in contrast to the *δ*^13^C values of the same region which were higher (−14.84‰) than the values of the other areas.

As it is previously reported, ref. [37,38,39] the carbon and nitrogen stable isotopes depend on nutrient uptake, digestion and metabolism of the organisms. Hence, the diet of each organism can explain the differences in these results. The *δ*^34^S_V-CDT_ ranged from 4.78‰ to 16.04‰ which are typical values for marine fish. Similar *δ*^34^S_V-CDT_ (4.6–20.1‰) have been reported from Sayle et al. (2013) [40]. Moreover, the study of Chen at al. [36] conclude that relatively higher values of *δ*^13^C_V-PDB_ (‰) could be used to discriminate farmed mullet Roes from wild-caught mullets. The authors claim that farmed mullets were fed with commercial fish feeds, which usually contain ingredients including fishmeal, plant-based proteins and other nutrients with soybean meal being often used to substitute fishmeal in commercial fish feeds to reduce manufacturing costs. In the same study, it is mentioned that free-living mullets since they are omnivores show a wide range of *δ*^13^C and *δ*^15^N values in their roes among individuals. Nevertheless, the stable isotopes of the mullets are affected by their feeding habitats and geographical locations. As it is presented in Figure 1, the areas in Greece where the mullets are fished are the Amvrakikos Gulf (Preveza) and the Messolongi–Aitoliko Lagoons. Both these aquatic ecosystems are quite closed, forcing the free-living mullets to specific food sources, affecting this way the spectrum of the isotopic values.

### 3.2. Isotopic Fingerprints Data and Decision Rules to Determine the Geographical Origin of the Bottagra PDO Product

**Figure 3:**
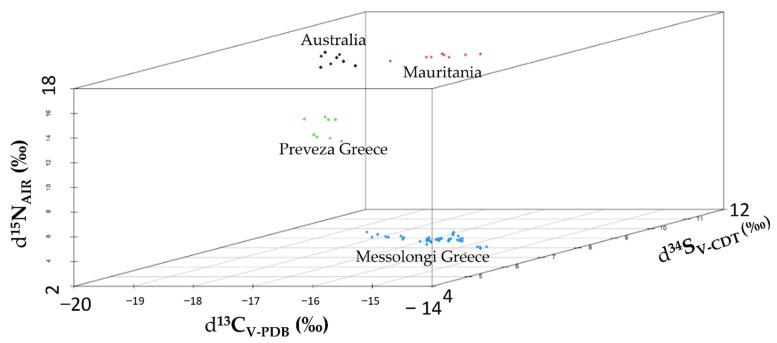
Results for Bottarga δ^15^N_AIR_ (‰), δ^13^C_V-PDB_ and δ^34^S_V-CDT_ (‰) mean values in a 3-dimension (3D) Scatterplot.

The calculated *δ* values for *δ*^15^N_AIR_ (‰), *δ*^13^C_V-PDB_ (‰) and *δ*^34^S_V-CDT_ (‰) are presented in a 3-dimension scatter plot (Figure 3). This figure clearly shows that the lower values of *δ*^15^N_AIR_ (‰) and *δ*^34^S_V-CDT_ (‰) can be used to discriminate the geographical origin of the Greek Bottarga Products. It is quite important to clarify why there are such low *δ* values, especially for sulphur, in the Messolongi mullet samples. Messolongi–Aitoliko Lagoons is a shallow costal area connected with two narrow openings in the north side with Messolongi–Aitoliko Lagoons and in the south with Gulf of Patras allowing seawater influx in the lagoons. In addition, the Acheloos and Evinos rivers have also participated in the formation of the lagoons through the deposition of sediment. Towns and highly cultivated agricultural areas in the vicinity of the lagoons produce an influx of sewage and irrigation run-off rich in nutrients. The influx of nutrients causes a high oxygen demand during decomposition of the organic matter. Moreover, large gypsum (CaSO_4_ 2H_2_O) deposits to the north-west of the Messolongi–Aitoliko Lagoons generate hydrogen sulphide in the hypolimnion (gypsum dissolves in water to produce sulphate ions (SO_4_)^−2^ which under anaerobic conditions are reduced to elemental sulphur and hydrogen sulphide). The organic and inorganic material rich in sulphur compounds is broken down by bacteria present on the lagoon bottom producing sulphides. The bacteria under anaerobic conditions produce S^−2^, HS^−^ and H_2_S with the reduction of sulphur. Hydrogen sulphide can either be oxidised to elemental sulphur by sulphur-bacteria or be released into the surrounding water [41,42]. According to Winner et. al., irradiated plants with roots immersed in (HSO_3_)^−^ and (SO_4_)^−2^ solutions at concentrations found in nature emit H_2_S from their leaves, and the emitted H_2_S contained more ^32^S and less ^34^S namely lower *δ*^34^S_V-CDT_ values in comparison to irradiation solutions [43].

The forementioned reasons explain the great difference of Messolongi *δ*^34^S_V-CDT_ values from the corresponding values of the other areas, helping to the authentication of bottarga geographical origin.

Probably the increased concentration of diluted H_2_S in the lagoon’s water has as result fishes that live in the lagoon and fishery products present low *δ*^34^S_V-CDT_ values. This explains the great difference in *δ*^34^S_V-CDT_ values between bottarga from Messolongi ‘Avgotaracho Messolongiou’ and bottarga from other areas examined. The results of this stable isotope analysis suggest that the geographical origin discrimination of bottarga products is feasible mainly to sulphur isotopes ratio.

**Figure 4:**
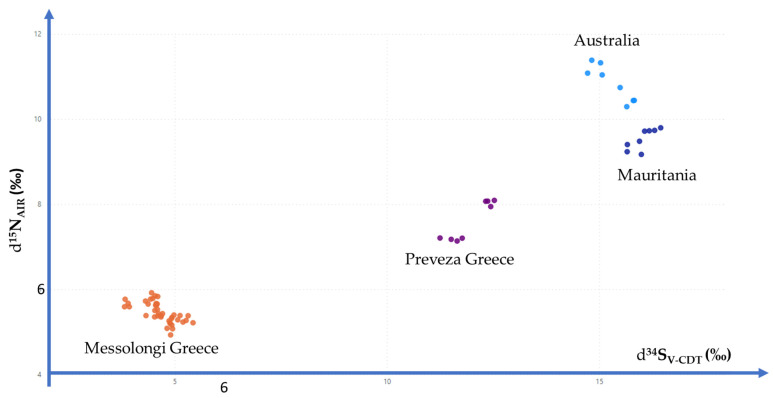
Results for Bottarga δ^15^N_AIR_ (‰) and δ^34^S_V-CDT_ (‰) in a 2-dimension scatterplot.

**Figure 5:**
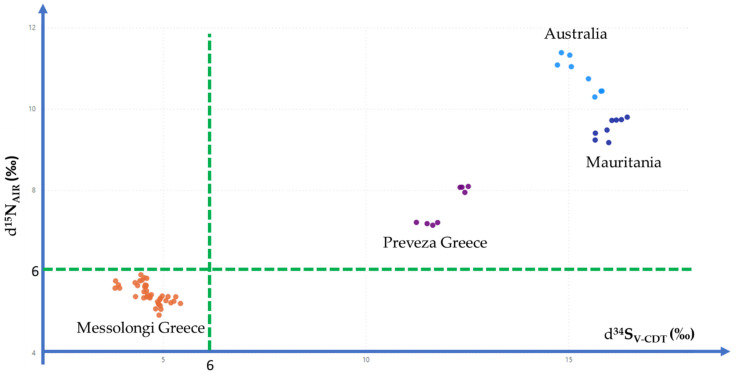
Decision criterion to determine geographic origin of the Greek Bottarga (Fish—Roe) Registered PDO ‘Avgotaracho Messolongiou’.

It is even easier if a 2-dimension scatter plot is used to present δ values. For example, Figure 4 presents *δ*^15^N_AIR_ (‰) values versus δ^34^S_V-CDT_ (‰) values. At a first glance, it seems quite simple and straightforward to use these *δ* to come up with a simple decision rule to examine if an examined bottarga sample comes from the registered area of the Greek PDO product ‘Avgotaracho Messolongiou’ by setting *δ*^15^N_AIR_ (‰) < 6.00 and at the same time *δ*^34^S_V-CDT_ (‰) < 6.00 (Figure 5). However, decision rules to discriminate geographical origin require extended research and comparisons with all different production areas.

## 4. Conclusions

Stable isotope analysis has been used in many products to authenticate their geographical origin. However, there are only a few studies that have been conducted for seafood origin and especially for mullet roes and bottarga products. This study tried to investigate the geographical origin of different bottarga products by means of verifying the authenticity of the Greek Bottarga product named ‘Avgotaracho Messolongiou’ the only Greek product registered as a Protected Designations of Origin (PDO). The results of the stable isotope analysis suggest that the geographical discrimination is feasible mainly to sulphur isotopes. The conducted analysis showed that δ^34^S_V-CDT_ (‰) values for the samples coming from Messolongi are significantly lower than the δ values for the other samples. Low δ^34^S_V-CDT_ (‰) values are attributed to high H_2_S concentration that the Messolongi–Aitoliko Lagoons’ bottom layer present. This special condition affects the ratio of sulphur isotopes, providing a base condition to create a decision rule to discriminate bottarga samples coming from this region.

Although further research is needed in order to include more areas producing such products, this study is the first to provide results to establish a decision rule to discriminate bottarga products.

## Figures and Tables

**Table 1:** Bottarga analysis results (mean δ values and s.d.).

Code	Area	Mean d^15^N_AIR_ (‰)	Mean d^13^C_V-PDB_ (‰)	Mean d^34^S_V-CDT_ (‰)
A1	Mauritania	9.53 ± 0,24	−19.02 ± 0.26	16.03 ± 0.89
A2	Australia	10.84 ± 0.42	−18.03 ± 0.59	15.30 ± 0.44
A3	Preveza Greece	7.61 ± 0.46	−18.01± 0.37	11.98 ± 0.49
A4	Messolongi Greece	5.45 ± 0.24	−14.82 ± 0.51	4.67 ± 0.38

**Table 2:** One way ANOVA analysis results (mean values and standard error).

*δ* (‰)	Area	N	Mean *δ* (‰)	Std. Error	ANOVA F Value	Sig.
d^15^N_AIR_ (‰)	Mauritania	8	9.53	0.09	985.040	0.000
	Australia	8	10.84	0.15		
	Preveza Greece	8	7.61	0.16		
	Messolongi Greece	40	5.45	0.04		
d^13^C_V-PDB_ (‰)	Mauritania	8	−19.02	0.09	273.388	0.000
	Australia	8	−18.03	0.21		
	Preveza Greece	8	−18.01	0.13		
	Messolongi Greece	40	−14.82	0.08		
d^34^S_V-CDT_ (‰)	Mauritania	8	16.03	0.10	3218.276	0.000
	Australia	8	15.30	0.16		
	Preveza Greece	8	11.98	0.17		
	Messolongi Greece	40	4.67	0.06		

## Data Availability

Data is contained within the article.
